# Predictors of Quality of Life among Parents of Children with Chronic Diseases: A Cross-Sectional Study

**DOI:** 10.3390/healthcare8040456

**Published:** 2020-11-03

**Authors:** Filiberto Toledano-Toledano, José Moral de la Rubia, Laura A. Nabors, Miriam Teresa Domínguez-Guedea, Guillermo Salinas Escudero, Eduardo Rocha Pérez, David Luna, Ahidée Leyva López

**Affiliations:** 1 Evidence-Based Medicine Research Unit, Hospital Infantil de Mexico Federico Gómez, National Institute of Health, Márquez 162, Doctores, Cuauhtémoc, Mexico City 06720, Mexico; 2 Facultad de Psicología, Universidad Autónoma de Nuevo León, Carlos Canseco, 110, Esq. Aguirre Pequeño, Col. Mitras Centro, Monterrey 64460, Mexico; jose_moral@hotmail.com; 3 School of Human Services, College of Education, Criminal Justice, and Human Services, University of Cincinnati, Cincinnati, OH 45221-0068, USA; naborsla@ucmail.uc.edu; 4 Department of Psychology and Communication Sciences, University of Sonora, Blvd. Luis Encinas y Rosales, Col. Centro S/N Hermosillo, Sonora 83000, Mexico; miriamd@sociales.uson.mx; 5 Centro de Estudios Económicos y Sociales en Salud, Hospital Infantil de Mexico Federico Gómez, National Institute of Health, Márquez 162, Doctores, Cuauhtémoc, Mexico City 06720, Mexico; guillermosalinas@yahoo.com; 6 Servicio Nacional de Sanidad, Inocuidad y Calidad Agroalimentaria (Senasica), Anillo Perif. 5010, Insurgentes Cuicuilco, Coyoacán, Mexico City 04530, Mexico; eduar_ronaldo15@hotmail.com; 7 Comisión Nacional de Arbitraje Médico, Mitla No. 250-8° Piso, esq. Eje 5 Sur (Eugenia), Vertiz Narvarte, Benito Juárez, Mexico City 03020, Mexico; xeurop@hotmail.com; 8 Centro de Investigación en Salud Poblacional, Instituto Nacional de Salud Pública, Cuernavaca Morelos 62100, Mexico; leyvalop@insp.mx

**Keywords:** quality of life, children, chronic diseases, family caregivers, psychosocial variables, sociodemographic factors, resilience, depression, family functioning, Mexico City

## Abstract

Quality of life (QOL) is a key aspect of the health care process for children with chronic diseases and their families. Although clinical evidence regarding the impact of chronic disease on children exists, few studies have evaluated the effects of the interaction between sociodemographic and psychosocial factors on the family caregiver’s QOL, indicating a significant gap in the research literature. The present study aimed to identify the predictors of the QOL of parents of children with chronic diseases. Three parental sociodemographic predictors (age, schooling, and family income) and four psychosocial predictors (family functioning, social support, depression, and resilience) were examined. In this cross-sectional study, 416 parents of children with chronic diseases who were hospitalized at a National Institute of Health in Mexico City were interviewed. The participants completed a sociodemographic variables questionnaire (Q-SV) designed for research on family caregivers of children with chronic disease. The predicted variable was assessed through the World Health Organization Quality of Life Questionnaire. The four psychosocial predictors were assessed through the Family Functioning Scale, Social Support Networks Scale, Beck Depression Inventory, and Measurement Scale of Resilience. The regression model explained 42% of the variance in parents’ QOL. The predictors with positive weights included age, schooling, monthly family income, family functioning, social support networks, and parental resilience. The predictors with negative weights included depression. These findings suggest that strong social relationships, a positive family environment, family cohesion, personal resilience, low levels of depression, and a family income twice the minimum wage are variables associated with better parental QOL.

## 1. Introduction

In 2008, a consensus definition of pediatric chronic disease was achieved. It consists of four criteria: a disease or condition in childhood is classified as chronic if the person is 0 to 18 years old; the diagnosis is based on a valid and reliable professional standard, such as the International Classification of Diseases of the World Health Organization; the disease is not curable at the present time or is very resistant to treatment, e.g., cancer, nephrotic syndrome or asthma; and the disease is active, has been present for at least three months, and is expected to persist and/or recur intermittently [1]. Diabetes mellitus ranks first among chronic diseases in Mexico. Ischemic heart disease is in second place, and malignant tumors are third. These three diseases account for almost half of all cases of chronic diseases in Mexican adults [2]. Cancer ranks first among the chronic diseases that cause mortality in Mexican children and adolescents. However, asthma and obesity are the most common chronic diseases. Ischemic heart disease is not present in this population, but abnormal blood flow due to a congenital heart defect is [2,3].

The epidemiological profile in Mexico has shifted from problems of low weight, short stature, and infectious diseases in the first half of the 20th century to chronic diseases, including metabolic syndrome and obesity, in childhood. According to epidemiological studies, the health system is adjusting to this new reality [2]. However, a major problem is the cost of medical care, especially for chronic diseases. In Mexico, medical expenses are covered by social security administered by the Instituto Mexicano del Seguro Social (IMSS). This insurance scheme is linked to formal employment contracts. Because the informal economy is dominant in Mexico, access to this insurance is limited within the Mexican population. For its part, the Ministry of Health of the Government of Mexico provides health care services to the entire population without social security through first- and second-level health care hospitals and the National Institutes of Health. These services are available to low-income families, especially those in the informal economy.

Faced with this situation, in 2003, the Seguro Popular was created; however, its coverage was partial and of poor quality [4]. On 1 January 2020, Seguro Popular was replaced by the Health Institute for Well-being. This institute provides universal coverage for all people of Mexican nationality or residence. However, there are states in the Mexican Federation that have not welcomed this change due to political opposition, which has left a large sector of the population without health care [5].

Quality of life (QOL) is a key aspect of health care for children with chronic diseases and their families. Empirical evidence shows significant deterioration in the QOL of both patients with chronic diseases and their family caregivers [6,7,8]. The profile of the average family caregiver of a child with a chronic disease treated at a public health center in Mexico is a woman aged approximately 30 years, married, works as a homemaker, is a mother, has a primary education level and a low socioeconomic level, has been dedicated to caring for children for more than one year, and dedicates more than six hours per day to childcare tasks [9,10].

Caring for children diagnosed with chronic diseases is an emotionally draining task, and the scientific literature related to this line of research has highlighted that family caregivers of children with chronic diseases spend a considerable amount of time on patient caregiving tasks and must modify their daily life activities as a result [11,12,13]. Moreover, studies have shown that as a child’s illness progresses, both the child and his or her caregivers experience a deterioration in QOL and a significant decrease in their physical, psychological, social, and economic well-being [14,15,16,17]. Therefore, some studies have aimed to assess the psychosocial factors associated with the QOL of family caregivers within the context of chronic diseases [8,13,18,19,20,21].

There are many factors that influence caregivers’ QOL. Several demographic factors have been highlighted for both children and caregivers. Among the main child sociodemographic variables associated with a higher QOL of the family caregiver are older age [22], male gender [23], an established medical diagnosis with a good prognosis [24], positive evolution and greater control of the disease [25,26], type of noninvasive treatment [27], and adaptive psychosocial functioning [28,29]. In addition, empirical evidence has shown that the main sociodemographic variables of caregivers that affect their QOL include male gender [30], older age [31], higher levels of schooling and income [32,33], being married [34], decreased caregiving time [35,36], reduced family caregiver burden [13], a shorter caregiving duration [37], less financial impact [38], a professional occupation [39], and greater religiosity or spirituality [40].

However, psychosocial variables seem to have a greater influence on caregivers’ QOL than sociodemographic variables [8,36,41,42,43,44,45,46,47,48]. Recent empirical findings show that family caregivers’ QOL is related to psychosocial variables, such as their parental stress [22,41], social support [25,38,42], family support [43], adaptation to the disease [44], depression [6,16], anxiety [20,36], coping styles [45], caregiver burden [46], self-perceived health [47], family functioning [8], and resilience [48].

These findings seem to confirm that the impact of caregiving during a child’s chronic disease has psychosocial effects on the QOL of family caregivers [6,7,8,14,15,16,17,49]. Despite these findings, few studies have examined the effects of both sociodemographic and psychosocial variables on the QOL of parents of children with chronic diseases in the social and cultural contexts of families in Mexico. Given the importance of contextualizing the extant literature within the psychosociocultural context of the Mexican family to verify similarities and reveal differences, the present study sought to identify which sociodemographic variables (age, schooling, and family income) and psychosocial variables (family functioning, family support, support networks, depression, resilience, caregiver burden, parental stress, and conservatism in relation to traditional Latin culture) are essential contributors to the QOL of parents of children with chronic diseases in the context of a public hospital in Mexico City. As complementary data, the relationship between children’s clinical variables (type of diagnosis, time since diagnosis, and length of hospitalization) and parents’ QOL was also analyzed.

Given the exploratory nature of this study, no hypothetical model to predict parents’ QOL was proposed or tested. Nonetheless, from reviewed studies conducted in nine developed countries (the Netherlands, Canada, the USA, the UK, Japan, South Korea, France, Greece, and Poland) and seven developing countries (Turkey, Mexico, Brazil, China, Colombia, India, and Nigeria), we expected that an older age among young and middle-aged parents [31], a higher level of schooling [32], and a higher family income [32], as numeric sociodemographic variables, would have a moderate effect on higher parental QOL. In addition, we hypothesized that better family functioning [8], more committed support networks [25,38,42,43], an absence or minimum of depressive symptomatology [6,16] or anxiety [20,37] and a higher level of resilience [48], as psychosocial variables, would have a large effect. Regarding the other variables included in this study, higher levels of caregiver burden and parental stress were expected to be associated with a lower QOL, since they involve negative feelings, while higher levels of well-being and family support were expected to be associated with a higher QOL, since they involve positive feelings [50]. The relationship between conservatism in relation to traditional Latin culture and QOL might be spurious and could be mediated by a low social status or low levels of schooling [51]. Notably, these predictive factors were proposed based on studies performed in populations of family caregivers who cared for children with chronic diseases, that is, studies carried out in pediatric settings. Finally, a significant association was expected between a lower parental QOL and the type of diagnosis (e.g., a disease with a poor prognosis requiring invasive treatments) [24,25,26], a shorter time since diagnosis [52], and a longer duration of hospitalization [53].

## 2. Materials and Methods

### 2.1. Study Design

Given its objectives, this study was descriptive and correlational/predictive; thus, the study was nonexperimental, and no variable was manipulated or controlled. The design was cross-sectional since the data were collected from the participants at a single time point.

### 2.2. Ethical Considerations

This study is part of “Research Project HIM/2013/019/SSA.1141 Measurement and assessment of resilience in pediatric chronic disease,” which was approved on 16 December 2013, by the Research, Ethics and Biosafety Commissions of the Hospital Infantil de Mexico Federico Gómez National Institute of Health, in Mexico City. While conducting this study, the ethical rules and considerations for research with humans currently enforced in Mexico [54], and those outlined by the American Psychological Association [55] were followed. All family caregivers were informed of the objectives and scope of the research and their rights according to the Helsinki Declaration [56]. The caregivers who agreed to participate in the study signed an informed consent letter. Participation in this study was voluntary and did not involve payment.

### 2.3. Sample

A nonprobability sampling technique was used to recruit a total of 416 voluntary participants who were parents of children with chronic diseases hospitalized at the Hospital Infantil de Mexico Federico Gómez National Institute of Health in Mexico City. The inclusion criteria for this study were as follows: a family caregiver (father or mother) of a child hospitalized at the institution with a chronic disease who was over 18 years of age and who had read, understood, and signed the informed consent form for the present research. The study included children with a wide variety of chronic childhood diseases; therefore, the only criterion regarding the process or stage of the disease was that it was in the active phase and severe enough to require hospitalization.

To estimate a linear regression model with a maximum of 11 variables with an effect size of at least R^2^ = 0.25, a significance level of 0.05, and a power of 0.99, the minimum sample size required was 111 participants. The sample size was approximately four times the minimum required, and there were more than 35 participants per variable.

### 2.4. Self-Assessment Instruments

The measurement instruments used in the present study were selected because they were developed in Mexico or adapted for the Mexican population. They have shown good or excellent overall internal consistency reliability and factors that are at least acceptable, a stable factorial structure, and evidence of construct validity; the exception is the Sociodemographic-Variable Questionnaire, for which only content validity has been established.

The *Sociodemographic Variables Questionnaire (Q-SV) for research on family caregivers of children with chronic disease*, by Toledano-Toledano et al. [57], contains 17 items that evaluate the following social, family, and clinical variables: age and gender of the patient and caregiver; patient’s diagnosis, duration of hospitalization and time since diagnosis; and family caregiver’s relationship to the patient (mother, father, other family member), education level (no schooling, primary, secondary, preparatory, bachelor’s degree, postgraduate), occupation (homemaker, manual laborer, merchant, employee, student, pensioner, unemployed), marital status (married, living with significant other, separated, divorced, single mother or father, widow/widower), number of years with partner, number of children, type of family (nuclear, semiextended, extended, single parent), family life stage (with small children, with school-age children, with adult children), social support networks (family, friends, religion, institutions, government), religion (Catholic, Christian, none), and monthly income.

*The World Health Organization Quality of Life Questionnaire*, developed by the WHOQOL Group [58], has been validated in the Mexican population [59]. This self-report instrument comprises 26 items with five response options answered on a Likert scale from 1 (very dissatisfied) to 5 (very satisfied). The items are distributed in the following four domains: physical health (e.g., “To what extent do you feel that physical pain prevents you from doing what you need to do?”), psychological health (e.g., “How much do you enjoy life?”), social relationships (e.g., “How satisfied are you with your personal relationships?”), and environment (e.g., “How safe do you feel in your daily life?”).

The *Family Functioning Scale* has been validated in the Mexican population [60]. This scale comprises 22 items with five response options, i.e., 1 (never) to 5 (always), that are distributed in the following four domains: positive family environment (e.g., “My partner and I agree on the rules and limits set for our children”), cohesion (e.g., “In my family, we like doing things together”), hostility/conflict avoidance (e.g., “In my family, we say one thing and do another”), and problems with rules and expressing emotions (e.g., “In my family, nobody follows the rules set by the parents”).

The *Social Support Networks Scale* [61] was validated in family caregivers of children with cancer by Toledano-Toledano et al. [62], and was also used in the present study. This scale is a self-report instrument comprising 45 items with five response options ranging from 1 (strongly disagree) to 5 (strongly agree) that are distributed in the following five factors: support from friends (e.g., “My friends and I are very important to each other”), family support (e.g., “We are a very close family”), lack of support (e.g., “My family and I cannot spend too much time together because we quarrel”), religious support (e.g., “When I have problems, I go to church”), and support from neighbors (e.g., “I know my neighbors will help me if I ask them to”).

The *Beck Depression Inventory* [63] has been validated with Mexican family caregivers of children with chronic diseases [16]. This instrument comprises 21 items distributed in two domains, i.e., cognitive-affective (e.g., sadness) and somatic (e.g., crying) factors, with four response options for each item (ranging from 0 to 3).

The *Measurement Scale of Resilience in Mexicans* (RESI-M) [64] was validated in family caregivers of children with cancer by Toledano-Toledano et al. [65]. This self-report instrument comprises 43 items, each with four response options ranging from 1 (totally disagree) to 4 (totally agree). The items are distributed in the following five domains: strength and self-confidence (e.g., “What happened to me in the past makes me feel confident when facing new challenges”), social competence (e.g., “I feel comfortable with other people”), family support (e.g., “I have a good relationship with my family”), social support (e.g., “I have some friends/relatives who truly care about me”), and structure (e.g., “Rules and routines make my life easier”).

The *Family Support Scale* (FSS) was developed in Mexico by Marin [66]. It is composed of 17 items that are rated on a four-point Likert-type scale (from 1 = “never” to 4 = “always”). This self-report instrument assesses the perceptions of family support among caregivers of pediatric patients with chronic diseases (e.g., “He/she lifts my spirits when I need it”). In a sample of 154 primary caregivers of children with cancer, the FSS had a one-factor structure, and its internal consistency reliability was excellent, with a Cronbach’s alpha of 0.96 [66].

The *Zarit Burden Interview* (ZBI) was developed by Zarit et al. [67] in the USA and validated in a Mexican population by Ramirez et al. [68]. It comprises 22 items that are rated on a five-point Likert-type scale (from 0 = “never” to 4 = “always”). The ZBI assesses caregiver burden. In a sample of 141 informal primary caregivers of children with chronic diseases, this self-report instrument presented a three-factor structure, i.e., impact of care on the caregiver (α = 0.88), caregiver-patient interpersonal relationship (α = 0.77), and self-efficacy expectations (α = 0.64). Its internal consistency reliability was excellent, with a Cronbach’s alpha equal to 0.90 [68].

The *Parental Stress Scale* (PSS) was developed in the USA by Berry and Jones [69] and was validated in Spain by Oronoz et al. [70]. The original version of the PSS comprises 17 items that are rated on a five-point Likert-type scale (from 1 = “totally disagree” to 5 = “totally agree”), and its internal consistency reliability was good, with a Cronbach’s alpha of 0.89 [70]. The PSS assesses the level of parental stress related to caring for children. Based on a sample of 211 married or cohabiting heterosexual individuals who had a baby, the PSS was reduced to 12 items (PSS-12) and showed a two-factor structure comprising rewards, with five items (α = 0.77), and stressors, with seven items (α = 0.76); its overall internal consistency reliability was good, with a Cronbach’s alpha of 0.83 [70].

The *Well-Being Index* of the World Health Organization was developed by Bech et al. [71] as a global measure of well-being for use in international studies. It comprises 10 items (e.g., “I feel energetic”) that are rated using a four-point Likert-type scale (from 0 = “never” to 3 = “all of the time”). Its overall internal consistency reliability is good, with a Cronbach’s alpha of 0.85 [71].

The *Historical Socio-Cultural Premises* Scale (HSCPS) was also used [51]. It was developed in Mexico to assess conservatism in relation to traditional Latin culture. It comprises 33 dichotomous items (1 = “no, I do not agree” and 2 = “yes, I agree”) and seven factors: machismo, affiliate obedience, female abnegation or marianismo, fear of authority, family status quo, family honor, and cultural rigidity. Its overall internal consistency reliability is good, with a Cronbach’s alpha of 0.88 [51].

### 2.5. Procedures

The participants received a letter of informed consent and were informed about the characteristics of the study, its benefits, the guarantees of anonymity and confidentiality, and the freedom to discontinue the study without institutional consequences for the participant or the patient. Only the parents who agreed to participate received instructions on how to respond to the sociodemographic data questionnaire. Subsequently, the participants answered all measurement instruments independently.

Data collection took place for approximately five months in 2019, and was performed by trained personnel in the Evidence-Based Medicine Research Unit of the National Institute of Health under the direction of the first author of this article. After delivering the questionnaire, the interviewer left the room unless the participant had a very low level of education and requested support to understand the questions, which occurred with only two participants. Approximately one hour after delivering the questionnaire, the interviewer returned to address any questions the participant had and to collect the self-report instruments. All of the participating family caregivers were parents of a child who had been diagnosed and was being treated for a chronic illness at the hospital. For this study, only one of the father caregivers, who usually took care of the child, was recruited; hence, the sample was composed mainly of women.

### 2.6. Data Analysis

The variables are described as the frequency (%), range (minimum and maximum), mean (M), and standard deviation (SD). To describe the sample, comparisons of sociodemographic variables between women and men were performed. The null hypothesis of equality in frequencies (gender ratio, occupation, marital status, type of family, and religious orientation) was tested using a binomial two-tailed test, Pearson’s right-tailed chi-square test or Fisher’s two-tailed exact test. The null hypothesis of equality was tested in mean ranks (schooling and income per month) using the Mann-Whitney two-tailed U-test and in arithmetic means (age and number of children) using Student’s two-tailed *t*-test.

Additionally, inferential tests were performed to identify the relationships among QOL, sociodemographic variables, and psychosocial variables, and a multivariate analysis using hierarchical regression was performed to observe the relationships between the two sets of predictive variables (sociodemographic and psychosocial variables) and the predicted variable (family caregiver QOL) [72]. Three sociodemographic variables were included in the model in the first step. The selected variables were those that had significant bivariate correlations with the predicted variable. For this purpose, the point-biserial correlation coefficient (r_pb_) was used for dichotomous variables, and Pearson’s product-moment correlation coefficient (r) was used for numeric variables [73]. Then, eight psychosocial variables were entered into the model in the second step, and a stepwise method was used to select these variables. Well-being was not included in the calculation of the model because its content was considered redundant or too strongly related to QOL and because the inclusion of well-being resulted in a less significant model.

Because we did not have a hypothetical structural model, neither path analysis nor structural equation modeling could be used. One of the advantages of these techniques is that they allow correlations between exogenous variables to be determined, while the linear regression exploratory technique requires low collinearity or independence between the predictors to avoid inflation of the explained variance in the criterion variable. Consequently, the collinearity between the predictors was examined. This entailed the use of an index of tolerance (TOL) and the variance inflation factor (VIF). TOL ≥ 0.80 and VIF ≥ 1.25 indicated low multicollinearity, while TOL < 0.40 and VIF > 2.5 indicated a concerning level of multicollinearity. Notably, the stepwise method facilitates the inclusion of predictors in the model with low collinearity, which is why this method was chosen [72].

In a linear regression model, the standardized regression weight (β) shows whether a predictor has an effect on the predicted variable. The following thresholds were used to interpret the size of the effect of each predictor on QOL: β < 0.10, trivial; 0.10 to 0.29, small; 0.30 to 0.49, moderate; 0.50 to 0.69, large; and ≥0.70, very large. Additionally, the coefficient of determination or squared multiple correlation coefficient (R^2^) shows whether the regression model has an effect on the predicted variable. The following thresholds were used to interpret the size of the effect of the model on QOL: R^2^ < 0.02, trivial; 0.02 to 0.12, small; 0.13 to 0.25, moderate; 0.26 to 0.49, large; and ≥0.50, very large [72,73]. The analyses were performed with SPSS 24 (IBM Corp, Armonk, NY, USA) and Excel 2007(Microsoft, Redmond, WA, USA), and nQuery 7 (Statsols, San Diego, CA, USA) was used to determine the sample size.

## 3. Results

### 3.1. Sample Description

The sample of 416 family caregivers included 340 women and 76 men. Only one main caregiver, either the mother or the father, was surveyed per child. All parents who were contacted to participate in the study expressed their desire to participate and signed the informed consent form; none of them refused to answer the questionnaires.

Table 1 displays the frequencies and percentages of the categorical variables, as well as the means and sample standard deviations of the quantitative variables in the pooled sample and in the samples of mothers and fathers. In addition, it shows probability values for hypotheses of equality between women and men in frequencies of nominal categorical variables, the mean ranks of ordinal categorical variables, and the arithmetic means of quantitative variables.

The mean age of the men was significantly higher (Student’s t (414) = 3.37, *p* = 0.001) than that of the women. The educational level was statistically equivalent between the genders (Mann-Whitney Z_U_ = −0.20, *p* = 0.840). In total, 44.2% of the parents reported having a primary education, 26% had a secondary education, 8.4% had a tertiary education, and 2.9% had a nonformal education. Regarding occupation (Pearson’s χ^2^[2, *N* = 416] = 172.26, asymptotic *p* < 0.001), 80.9% of the women performed unpaid work (i.e., their work efforts were exclusively dedicated to the home or studying) compared to 2.6% of the men; 15.9% of the women performed paid work, compared to 75% of the men; and 3.2% of the women were unemployed, compared to 22.4% of the men. There was also a difference in monthly family income (Mann-Whitney Z_U_ = −2.08, *p* = 0.038), with a lower average among the women than among the men. In total, 63.5% of the women and 50% of the men reported an income lower than 140 USD, 33.5% of the women and 44.7% of the men reported an income between 140 and 418 USD, and 3% of the women and 5.3% of the men reported an income greater than or equal to 419 USD. There was a significant difference in marital status (Fisher’s χ^2^[5, *N* = 415] = 13.07, 2-tailed exact *p* = 0.015). The only differing category in the pairwise comparisons using Bonferroni’s correction was single marital status (9.4% of the mothers versus 0% of the fathers). When this marital status category was excluded, there was no difference in the other five categories (Fisher’s χ^2^[4, *N* = 383] = 2.63, 2-tailed exact *p* = 0.604). The arithmetic mean number of children was 2.31, with no difference between women and men.

There was a difference between the genders in the type of family (Pearson’s χ^2^[4, *N* = 416] = 20.67, *p* < 0.001). A nuclear family (spouses and children living in their own home) was more common among the men (72.4%) than among the women (46.2%). In contrast, a single-parent family (one parent living with the children) was more common among the women (18.5) than among the men (2.64). For the three other family categories, there was no gender difference according to the pairwise comparisons with Bonferroni’s correction. In total, 15.9% of the participants reported having a semiextended family (nuclear family and one or more relatives living together), 10.3% of the participants reported having an extended family (nuclear family living with the family of origin of one of the spouses), and 7.2% of the participants reported having another type of family.

Table 2 shows that the parents had an average WHOQOL total score (M = 83.78, SD = 11.06) close to high, and the averages were particularly high for social relationships (M = 10.62, SD = 2.16) and the environment (M = 24.89, SD = 4.47). No participant reported a very low level of QOL (WHOQOL total scores from 26 to 46), and only 6.7% reported a low level (WHOQOL total scores from 47 to 67). In contrast, 59.1% reported a moderate level of QOL (WHOQOL total scores from 68 to 88), 33.2% reported a high level (WHOQOL total scores from 89 to 109), and 1% reported a very high level (WHOQOL total scores from 110 to 130). The average score in family support scale was also high (M = 59.44, SD = 10.14). The average scores were moderate for family functioning (M = 69.47, SD = 6.78), social support networks (M = 159.01, SD = 17.99), resilience (M = 133.75, SD = 16.13), and well-being (M = 18.20, SD = 5.19). On the other hand, the parental caregivers had low average scores for depression (M = 13.81, SD = 9.73), caregiver burden (M = 23.22, SD = 12.09), parental stress (M = 19.68, SD = 6.92), and cultural conservatism in relation to traditional Latin culture assessed by HSCPS (M = 49.47, SD = 6.15).

Regarding the overall internal consistency reliability of the measurement instruments, eight out of ten scales had an excellent level (Cronbach’s α ≥ 0.90), and the Parental Stress Scale and Historical Socio-Cultural Premises Scale had a good level (Cronbach’s α ≥ 0.80). Table 2 shows the Cronbach’s alpha values for both the scales and their factors.

Regarding the sociodemographic variables of the children, approximately half were girls (52.6%), and the other half were boys (48.4%). Their average age was 5.91 years (SD = 5.03), with a range from 1 to 17 years. The age distribution of these patients showed a long tail to the right (Z_Sk_ = 6.97 > 1.96). Thus, the values of the mode (Mo = 1 year, 24.8% of the children) and the median (Mdn = 4 years) were below the value of the arithmetic mean. On the other hand, the distribution profile presented a mild platykurtosis (Z_K_ = −2.46 < −1.96), that is, a higher concentration of values around the mean (shoulder area) than a normal distribution. Ages 1 to 6 years accumulated two thirds of the frequency, and ages 7 to 17 years one third.

The clinical variables of these pediatric patients are shown in Table 3. Most of the patients were being treated for an oncological disease (74%). Most of them had been hospitalized for a maximum of one week (63.2%) or one month (21.6%) at the time of the survey. The time elapsed since diagnosis varied from one week or less (26%) to ten years or more (10.3%), with a median of one year or less. The type of diagnosis, based on the six categories with more than 10 cases (ANOVA: F(5, 390) = 1.54, *p* = 0.176, η = 0.14), as well as the time elapsed since the diagnosis (r_S_ = −0.40, *p* = 0.411) and the length of hospitalization (r_S_ = 0.43, *p* = 0.384), were independent of the parents’ QOL total score.

### 3.2. Relationship between Sociodemographic and Psychosocial Factors and QOL

To analyze the multivariate relationship between the psychosocial and sociodemographic profiles and the burden perceived by the parents, a hierarchical regression was performed. The first set of variables included in the model comprised the following sociodemographic factors: age, schooling, and parents’ monthly family income. Caregivers’ schooling was dichotomized as 0 = no schooling or primary education and 1 = secondary education or higher, and family income was dichotomized as 0 = less than 2 minimum wages and 1 = 2 or more minimum wages. Primary schooling or no schooling and a family income below two minimum wages are clearly associated with precarious situations [74]. Hence, these cutoff points were used to dichotomize these two variables. These ordinal variables were dichotomized to allow the use of the r_pb_ and the introduction of the variables in the regression model. The gender of the parent (r_pb_(416) = −0.02, *p* = 0.675) and the number of children were independent of QOL (r(416) = −0.01, *p* = 0.928); therefore, these two sociodemographic variables were not included in the model.

The second set of variables entered into the model comprised eight psychosocial factors, including family functioning, social support networks, depression, and resilience. The other psychosocial variables had significant correlations with QOL but were excluded from the model using the stepwise method. The strength of the association was moderate for family support (r(416) = 0.34, *p* < 0.001) and weak for caregiver burden (r = −0.29, *p* < 0.001), parental stress (r(416) = −0.21, *p* < 0.001) and conservatism in relation to traditional Latin culture (r(416) = −0.13, *p* = 0.007). All these variables correspond to the total scores of their corresponding scales.

Table 4 shows the results of the hierarchical multiple regression analysis. The two sets of variables had significant weight for predicting the QOL of the parental caregivers (F(7, 408) = 62.54, *p* < 0.001) and explained 42% of the variance (adjusted R² = 0.42). The positive predictors of QOL included schooling (β = 0.20, *p* < 0.001), age (β = 0.14, *p* < 0.01), and family income (β = 0.13, *p* < 0.05), as well as resilience (β = 0.31, *p* < 0.01), social support networks (β = 0.14, *p* < 0.001), and family functioning (β = 0.09, *p* < 0.05). The negative predictor was depression (β = −0.29, *p* < 0.001). Five of the seven predictors showed low collinearity (TOL > 0.80 and VIF < 1.25). Social support networks (TOL = 0.76 and VIF = 1.32) and resilience (TOL = 0.70 and VIF = 1.43) were the variables with the highest collinearity, but their degree of collinearity was not concerning.

The well-being index had a moderate positive correlation (r(416) = 0.47, p < 0.001) but was not included in the model calculation. Its content was considered redundant or too closely related to QOL. If it had been included, it would have replaced family functioning as a predictor (β = 0.16, *p* = 0.001). The other predictors would have been the same, except for income, which would have been excluded. The explained variance would have been very similar (42.9%). The previous model was considered more significant at the level of interpretation; therefore, the latter model was discarded.

## 4. Discussion

This study analyzed the sociodemographic and psychosocial variables that were related to and predicted the QOL of parents of children with chronic diseases who were hospitalized at the National Health Institute of Mexico City. The main findings suggest that although both types of variables affected QOL, psychosocial variables had a greater impact.

The parents included in this study were predominantly young adult women who had a primary or secondary education, were Catholic, and had a low income. These characteristics match those reported in previous studies [9,75]. The patient characteristics included a diagnosis of cancer (three-quarters of the patients) or another chronic disease (including medical complications of Down syndrome), a time since diagnosis of over three years and a length of hospitalization of over one month.

In the present study, QOL seemed to be relatively good in general, primarily in the social relationships and environment domains, and there were low levels of parental stress and depression. These results contrast those reported in previous studies [6,7,63], which indicated a significant decrease in the QOL of family caregivers of children with chronic diseases. As in previous research [46,76], this study found that the physical health of caregivers was the domain that was most affected by caring for children. Specifically, Rubira et al. [76] stated that the negative effects of caregiving reflect poor perceived health rather than actual deterioration in this domain.

Notably, the parents of children with chronic diseases in the present study had more traits associated with resilience in the face of the disease than other studies have reported. For example, the parents showed strengths such as self-confidence, family support, social support, and adherence to rules and activities that allowed them to organize their daily life. High scores for perceived social support from family and friends were also observed. This trend was reflected in the high family support and family functioning scores observed in the domains of positive family environment and cohesion.

The hypotheses formulated regarding the relationship between QOL and psychosocial variables were confirmed. The associations of QOL with resilience [77], support networks [25,38,42], well-being [50], family functioning [8], and family support [43] were positive. In contrast, the associations of QOL with depressive symptoms [6,16], caregiver burden [46], and parental stress [20,36] were negative. However, there were four variables with true predictive power for QOL: high levels of resilience, social support, and family functioning and low levels of depressive symptoms predicted better QOL among the parental caregivers. These findings are comparable to those described in other studies in similar contexts [38,43]. The results of this study suggest that the psychological well-being of parents is related to social and family support and the ability to overcome adversity.

Conservatism in relation to traditional Latin culture showed a negative association with QOL. This inverse correlation is expected to occur due to the higher level of conservatism among people with low socioeconomic status and lower levels of education [51]. Both sociodemographic variables presented negative correlations with cultural conservatism and positive correlations with QOL. In addition, when one of these two sociodemographic variables was partialized, the correlation between cultural conservatism and QOL ceased to be significant, which indicates that this relation was spurious and was mediated by the sociodemographic variables. Consequently, this psychocultural variable was not entered as a predictor in the regression model [73].

Regarding the sociodemographic variables, higher levels of education and income were found to predict and affect the QOL of the parental caregivers. These results confirm expectations based on previous findings reported by Bellin et al. [32] and Vanz et al. [33] regarding the relationship between these variables. Additionally, higher parental age was associated with better QOL, as anticipated [33]. However, our study showed that the gender of the parents was associated with participation in the care of the child but not with their QOL, consistent with previous research [78].

Although the present findings are generally consistent with those reported in other studies conducted under diverse conditions, notably in the Mexico City context, high levels of resilience, social support, and family functioning were significantly associated with better QOL.

Based on the present results, interventions to improve the QOL of parental caregivers could focus on psychosocial variables, which are easier to modify than sociodemographic variables. Specifically, interventions implemented by health psychologists or nurses could focus on the development of resilience skills, such as strength and self-confidence, social competence, family support, social support and personal structure [79].

The chronic diseases of the pediatric patients varied; cancer was the most frequently occurring disease, consistent with the epidemiological data of the hospital in which the research was conducted. For this reason, the population was defined as parents of children with chronic diseases. Consequently, the data were analyzed as a single set. This approach is neither new nor exclusive to this study [13,15,16,17,19,20,28,36,40,42,44]. In future studies, analyses can be performed by diagnostic subgroups to deepen the understanding of the similarities and differences between subgroups.

Because they are complementary data, the associations between the clinical variables of the children with chronic diseases in the present sample and the QOL of their parental caregivers are shown. The three clinical variables included in this study were independent of the parents’ QOL. Consequently, the expectation of a significant association between parental QOL and type of diagnosis [24,25,26], time since diagnosis [52] and length of hospitalization [53] were not fulfilled and could not have been entered into a predictive model using another statistical technique, such as binary logistic regression. However, there are other variables, such as disease prognosis [25], disease control [26] and type of treatment [26], which could be important predictors of family caregiver QOL and could be considered in future studies. The dichotomous variable being hospitalized or not could also be another significant predictive factor, since the hospitalization of a child is a highly stressful event that entails many changes in family routine and organization [80]. In addition to the clinical variables, another block of variables that could be included in future studies for predicting the QOL of the family caregiver is the age and gender of the children [81].

One recommendation for future studies is the use of longitudinal research designs that allow assessments to be made of the temporal evolution of the relationships detected and an estimation of the prevalence of the results obtained. Moreover, it could be useful to include more diverse groups of family caregivers from different institutions to categorize their main psychological and demographic characteristics and explore the associations with more specific interpersonal skills and protective factors.

A limitation of this study is the use of nonprobabilistic sampling; thus, inferences should be made with caution. Consequently, the conclusions of this study could be adopted as hypotheses or comparative data in similar or comparable populations, that is, other parents of children with chronic diseases who are hospitalized. However, efforts were made to prevent biases during the data collection and analysis processes. In addition, the sample size was sufficient and large. For a regression model with seven predictors with a large effect size (approximately four-tenths of the variance in the criterion variable was explained), a significance level of 0.05 and a power of 0.99, the minimum sample size required was 49 participants. The actual sample size was nine times larger than the minimum required, with approximately 60 participants per predictor and more than 400 participants in total. Additionally, the internal consistency reliability of the measurement instruments was excellent or good. Moreover, these tools have been validated in the population under study, except for the HSCPS, which was created in Mexico. Another limitation is the nonexperimental and cross-sectional design, which did not allow causal inferences; hence, only correlations and predictions were discussed in this paper. A third limitation is the self-reported nature of the data, which resulted in a lack of more objective behavioral or cognitive data.

## 5. Conclusions

In this sample of parents of children with chronic illnesses who were hospitalized, a secondary or tertiary education, older age (relative to an average age of approximately 32 years), and a monthly family income over 279.36 USD predicted higher levels of parental QOL. The effect sizes of each of the three sociodemographic variables and the entire set of psychosocial variables were small. A higher level of resilience predicted higher parental QOL with a moderate effect size; a lower frequency of depressive symptoms and greater social support predicted higher parental QOL with small effect sizes; and better family functioning predicted higher parental QOL with a trivial effect size. The selected sociodemographic and psychosocial variables explained more than two-fifths of the variance in QOL. Since the multicollinearity between the variables was low and there was no inflation of the variance as a result of multicollinearity, it can be said that these variables had a large effect size.

In the present data, none of the parents reported a very low QOL level, which is a positive finding for hospital care teams. Six out of ten parents rated their QOL as moderate, and three out of ten rated it as high. However, one in ten did report a low QOL level. All self-assessments are based on a process of comparison [82]. Upon considering why the self-assessment of QOL level was so positive among the parents, we conjectured that parents compare themselves to their hospitalized children, who suffer from a chronic illness that seriously affects their QOL. In this comparison, the parents are much better off and, consequently, their self-evaluation is positive.

Parents with a lower education level might benefit from working on aspects of resilience in group therapy. Likewise, the level of depressive symptoms in these parents must be assessed. In the case of mild or more severe depression, depressive episodes may be modifiable with group or individual psychotherapy.

## Figures and Tables

**Table 1:** Summary statistics of the parents’ sociodemographic variables.

Variable Label	Value Label	Pooled	Mother	Father	Statistical Test	*p*
		*n* (%) or M ± SD	*n* (%) or M ± SD	*n* (%) or M ± SD		
Gender and caregiver role	Female (mother)	340 (81.7%)	340 (100%)		B	<0.001
	Male (father)	76 (18.3%)		76 (100%)		
Schooling	No schooling	12 (2.9%)	11 (3.2%)	1 (1.3%)	MW Z_U_	0.840
	Primary	77 (18.5%)	62 (18.2%)	15 (19.7%)		
	Compul. secondary	184 (44.2%)	151 (44.4%)	33 (43.4%)		
	Higher secondary	108 (26%)	87 (25.6%)	21 (27.6%)		
	University	35 (8.4%)	29 (8.5%)	6 (7.9%)		
Occupation	Unpaid work	277 (66.6%) *	275 (80.9%)	2 (2.6%)	χ^2^ (2)	0.001
	Homemaker	274 (65.9%)	273 (80.3%)	1 (1.3%)		
	Student	3 (0.7%)	2 (0.6%)	1 (1.3%)		
	Paid work	111 (26.7%) *	54 (15.9%)	57 (75%)		
	Office employee	58 (13.9%)	34 (10%)	24 (31.6%)		
	Seller/merchant	39 (9.4%)	17 (5%)	22 (28.9%)		
	Laborer	14 (3.4%)	3 (0.9%)	11 (14.5%)		
	Unemployed	28 (6.7%) *	11 (3.2%)	17 (22.4%)		
Marital status	Married	169 (40.6%)	133 (39.2%)	36 (47.4%)	Fisher’s exact *p*	0.017
	Cohabitating	161 (38.8%)	127 (37.5%)	34 (44.7%)		
	Separated	37 (8.9%)	33 (9.7%)	4 (5.3%)		
	Single parent	32 (7.7%) *	32 (9.4%)	0 (0%)		
	Divorced	12 (2.9%)	10 (2.9%)	2 (2.6%)		
	Widowed	4 (1%)	4 (1.2%)	0 (0%)		
Income per month ^#^ (minimum wage)	<1 ≈ 140 USD	254 (61.1%)	216 (63.5%)	38 (50%)	MW Z_U_	0.038
	1 to 2 ≈ 279 USD	91 (21.9%)	69 (20.3%)	22 (28.9%)		
	2 to 3 ≈ 419 USD	57 (13.7%)	45 (13.2%)	12 (15.8%)		
	≥3	14 (3.3%)	10 (3%)	4 (5.3%)		
Religious orientation	Catholic Christian	340 (81.7%)	275(80.9%)	65 (85.5%)	χ^2^ (2)	0.135
	Non-Catholic Chr.	48 (11.6%)	44 (12.9%)	4 (5.3%)		
	No religion	28 (6.7%)	21 (6.2%)	7 (9.2%)		
Age (years)		31.67 ± 8.02	31.05±7.74	34.43 ± 8.72	*t*-test	0.001
N. of children		2.31 ± 1.19	2.31 ± 1.18	2.29 ± 1.20	*t*-test	0.898

Note. In the third column, the simple absolute frequency (n) and simple percentage (%) are shown for qualitative and ordinal variables, and the arithmetic mean ± sample standard deviation (M ± SD) is shown for quantitative variables. Statistical tests (fourth column) and probability values (fifth column): B = two-tailed probability value for the binomial test, χ^2^(df) = right-tailed probability value for Pearson’s chi-square test with degree of freedoms (df), Fisher’s exact p = two-tailed probability value for Fisher’s exact test, MW Z_U_ = two-tailed probability value for the Mann-Whitney standardized U statistic, and *t*-test = two-tailed probability value for Student’s *t*-test statistic assuming homogeneity of variances tested by Levene’s test. Sample size: *N* = 416. * Category with nonhomogeneous frequencies between both genders in the pairwise comparisons using Bonferroni’s correction for significance level. ^#^ In 2018, the general minimum wage per month was 2686.14 Mexican pesos. The exchange rate on 26 December 2019, was one Mexican peso = 0.052 USD.

**Table 2:** Descriptive factors/subscales and psychosocial variables.

Psychosocial Variable	Factor/Subscale	Items	Range	M	SD	Alpha
WHOQOL Questionnaire		26	58–117	83.78	11.06	0.90
(World Health	Physical health	7	11–33	20.75	3.17	0.71
Organization	Psychological health	6	9–23	16.92	2.52	0.69
Quality of Life	Social relationships	3	3–15	10.62	2.16	0.67
Questionnaire)	Environment	8	13–40	24.89	4.47	0.75
Family Support Scale		17	17–68	59.44	10.14	0.97
Family Functioning Scale		22	47–95	69.47	6.78	0.91
	Positive family environment	7	7–35	29.19	4.85	0.77
	Cohesion	5	6–25	20.00	3.76	0.79
	Hostility/conflict avoidance	5	5–23	9.43	3.36	0.68
	Rules/emotional expression	5	5–25	10.85	4.37	0.67
Social Support Networks Scale		45	13–82	159.01	17.99	0.90
	Support from friends	15	18–75	51.90	10.20	0.91
	Family support	15	23–75	63.02	9.43	0.91
	Lack of support	7	7–31	15.81	4.55	0.68
	Religious support	4	4–20	15.01	3.33	0.78
	Support from neighbors	4	4–20	13.66	2.91	0.60
Mexican Resilience Measurement Scale		43	83–172	133.75	16.13	0.95
	Strength and self-confidence	19	38–76	60.17	8.16	0.93
	Social competence	8	10–32	23.02	3.98	0.87
	Family support	6	6–24	19.95	3.13	0.87
	Social support	5	5–20	16.21	2.95	0.90
	Structure	5	5–20	14.37	2.46	0.76
Well-Being Index		10	0–30	18.20	5.19	0.90
Beck Depression Inventory		21	0–55	13.81	9.73	0.91
	Cognitive-affective	12	0–32	6.84	6.01	0.81
	Somatic	7	0–23	6.96	4.58	0.81
Zarit Burden Interview		22	0–88	23.22	12.09	0.90
Parental Stress Scale		12	5–60	19.68	6.92	0.90
	Stressors	5	7–35	12.75	5.42	0.76
	Rewards	7	5–25	6.93	2.47	0.83
HSCPS		33	33–66	49.47	6.15	0.85

Note. Range (minimum value and maximum value), items = number of items in each scale or factor, M = arithmetic mean, SD = sample standard deviation, alpha = Cronbach’s alpha coefficient. HSCPS = Sociocultural Historical Premises Scale. The first two items of the WHO Quality of Life Questionnaire were entered only into the calculation of the total score and were not included in the determination of factors since they are general or nonspecific items.

**Table 3:** Frequencies and percentages of the pediatric patients’ clinical variables and their association with parents’ QOL total scores.

Variable	Value	*n*	%	∑%	Coef.		*p*
Diagnosis	Cancer	308	74		η	0.14	0.176
	Abnormal blood flow due to a congenital heart defect	28	6.7				
	Nephrotic syndrome	21	5				
	End-stage renal disease	15	3.6				
	Tricuspid atresia	12	2.9				
	Asthma	12	2.9				
	Down syndrome *	9	2.2				
	Tetralogy of Fallot	4	1				
	HIV/AIDS	3	0.7				
	Cystic fibrosis	2	0.5				
	Organ transplant	2	0.5				
Hospitalization duration	One week or less	263	63.2	63.2	r_S_	−0.04	0.411
	One month or less	90	21.6	84.8			
	Six months or less	34	8.2	93			
	One year or less	14	3.4	96.4			
	More than one year	15	3.6	100			
Time elapsed since diagnosis	One week or less	108	26	26	r_S_	0.38	0.384
	Three months or less	47	11.3	37.3			
	Six months or less	52	12.5	49.8			
	One year or less	74	17.8	67.6			
	Three years or less	57	13.7	81.3			
	Five years or less	35	8.4	89.7			
	Ten years or less	43	10.3	100			

Note. Statistics: *n* = simple absolute frequency, % = simple percentage, ∑% = cumulative percentage. Coefficients of association: η = eta and *p* = right-tailed probability value for ANOVA’s F-statistic, with homogeneity of variance among six groups (with *n* > 10) tested by Levene’s test; r_S_ = Spearman’s rank-order correlation, and *p* = two-tailed probability value. Sample size: *N* = 416. * Down syndrome is a chromosomal condition (the presence of all or part of a third copy of chromosome 21) that is not classified as a chronic disease, but patients are referred to the chronic disease care unit when they have cardiac, digestive or metabolic disorders associated with this syndrome.

**Table 4:** Model of parental quality of life based on hierarchical regression.

Variables	r with PV	B	β	TOL	VIF	F(df1, df2)	R and R^2^ Values
Sociodemographic							
Schooling	0.22	4.59 ***	0.2	0.88	1.13	F(3, 412) = 13 ***	R = 0.29
							R² = 0.09
Age	0.14	0.19 **	0.14	0.96	1.04		Adj. R² = 0.08
Family income	0.2	3.66 *	0.13	0.88	1.14		ΔR² = 0.86 ***
Psychosocial							
Resilience	0.55	0.21 ***	0.31	0.7	1.43	F(7, 408) = 62.54 ***	R = 0.66
Social support networks	0.4	0.09 ***	0.14	0.76	1.32		R² = 0.43
Family functioning	0.24	0.14 *	0.09	0.89	1.12		Adj. R² = 0.42
Depression	−0.49	−0.33 ***	−0.29	0.81	1.23		ΔR² = 0.35 ***

Note. PV = predicted variable (family caregiver quality of life corresponding to WHOQOL Questionnaire total score). All predictors are sociodemographic or psychosocial variables of the parents: Family functioning (Family functioning scale total score), Social support networks (Social support networks scale total score), Depression (Beck Depression Inventory total score), and Resilience (Mexican Resilience Measurement Scale total score). Schooling (0 = no schooling or primary education and 1 = secondary or higher education). Family income (0 = < 2 minimum wage ≈ 279.36 USD and 1 = ≥ 2 minimum wage). The variables that were included in the calculation but were not entered in the model were family support (family support scale total score), caregiver burden (Zarit Burden Interview total score), parental stress (parental stress scale total score), and conservatism in relation to traditional Latin culture (HSCPS total score). r = Pearson product-moment correlation coefficient. B = raw regression coefficient. β = standardized regression coefficient. TOL = tolerance index. VIF = variance inflation factor. F = test statistic. df_1_ = degrees of freedom for the regression sum of squares. df_2_ = degrees of freedom for the residual sum of squares. R = multiple correlation coefficient. R^2^ = squared multiple correlation coefficient or the proportion of variance in the outcome variable explained by the predictors. Adjusted R² = adjusted squared multiple correlation coefficient or squared epsilon coefficient. ΔR² = quotient of the relationship between the increase in the residual sum of squares and the total sum of squares. Significance with a two-tailed test: * *p*-value ≤ 0.05, ** *p*-value ≤ 0.01, *** *p*-value ≤ 0.001. *N* = 416.
